# Police stops and adolescent sleep problems: findings from the UK millennium cohort study

**DOI:** 10.1111/jsr.13585

**Published:** 2022-03-14

**Authors:** Dylan B. Jackson, Alexander Testa

**Affiliations:** ^1^ Johns Hopkins Bloomberg School of Public Health Baltimore Maryland USA; ^2^ The University of Texas Health Science Center at Houston Houston Texas USA

**Keywords:** adolescents, health, police, sleep, UK, youth

## Abstract

Youth‐police contact has been shown to undermine various facets of adolescent health and well‐being. The present study extends this body of research by assessing the association between police stops and sleep problems among a large, representative sample of adolescent in the UK (*N* = 11,200). The findings reveal youth–police contact was associated with all facets of sleep examined, including shorter sleep durations, longer sleep latency, and frequent mid‐sleep awakenings. Ancillary analyses of a subsample of youth using daily time‐use diaries (TUDs) largely corroborate these findings in the case of short sleep durations. Additionally, the odds of experiencing multiple sleep problems were greatest among youth who were arrested/taken into custody, but significant nonetheless even in the absence of arrest. These findings indicate the need for strategies and interventions among both public health practitioners and law enforcement that mitigate the adverse repercussions of police stops for adolescent sleep health.

## 1 INTRODUCTION

Aggressive and unfair police treatment have been shown to undermine population health (DeVylder et al., 2017; Geller et al., 2014; Jackson et al., 2019; McFarland et al., 2019; Turney, 2021). Youth appear to be particularly vulnerable to the adverse health repercussions of proactive policing, as they can experience diminished self‐rated health (McFarland et al., 2019), heightened anxiety (Geller et al., 2014), post‐traumatic stress (Geller et al., 2014; Jackson et al., 2019), social stigma (Jackson et al., 2019), and depression (Turney, 2021) following police stops.

While research demonstrates the substantial costs of aggressive policing to youths’ health and wellbeing, one facet of health that is often overlooked in this body of work is sleep. Despite the potential for police stops to interfere with youth sleep, only one study has investigated this relationship. The findings from a sample of youth born in urban areas in the USA reveal that police stops characterised by officer intrusiveness were associated with lower sleep quality among youth, an association largely explained by the stigma and post‐traumatic stress engendered by the stop (Jackson et al., 2020). Recent findings among a nationally representative study in the USA also suggest a similar pattern in adults, who report sleep deprivation and more frequent trouble sleep following lifetime exposure to unfair police treatment (Testa et al., 2021). Despite the results of these two studies, there is no research to date examining whether the nexus between police stops and sleep generalises outside of the USA context.

Certainly, there are important differences in policing in the USA and other developed democracies such as the UK, which could have meaningful implications for whether or how police contact might be connected to sleep problems among citizens. Policing in the UK is highly centralised with ~50 police forces across the UK, compared to the decentralised structure of >18,000 mostly local agencies across the USA (Evans, 2007). Policing in the UK also has a greater community‐oriented focus, with the cultural emphasis on serving with the consent of the public. Relatedly, due to the centralised nature of policing in the UK, there is greater governmental oversight (Evans, 2007; Sherman, 2020). In contrast, the USA has adopted a more proactive and militarised policing strategy, which is characterised by the use of pre‐emptive stops and the public display of firearms and other weapons, attire, and tactics previously reserved for the military (Mummolo, 2018; Steidley & Ramey, 2019; Weisburd & Majmundar, 2018). This is contrasted with policing in the UK where police often do not carry firearms, but instead rely more commonly on tasers (Ariel et al., 2019). In part, this may contribute to less lethal use of force incidents in the UK relative to the USA (Edwards et al., 2019; Lartey, 2015). Accordingly, due to the more decentralised and proactive nature of policing in the USA, it is possible that the repercussions of police contact for youth sleep in the UK may not echo findings in United States data (Jackson et al., 2020; Testa et al., 2021).

Alternatively, there is also reason to believe that police contact in the UK could have similarly harmful repercussions for the sleep of youth. For example, use of force by UK officers may still represent a threat in the minds of youth, as there have been publicised use of force cases with tasers documented against frontline officers in the UK (Ariel et al., 2019). Recent data also suggest patterns of racial disparity with stops and searches, as well as general use of force, disproportionately used against Black individuals in the UK (Home Office, 2021; Vomfell & Stewart, 2021). Substantial numbers of UK youth, moreover, distrust or fear the police (Travis, 2014). Although less research has been conducted on the consequences of police contact in the UK, recent studies intimate that police contact in the UK context carry negative repercussions for youth health outcomes in a similar fashion to the USA (Jackson et al., 2022; Jackson et al., 2021).

Despite the possibility of a connection between police contact and youth sleep problems in the UK, research has yet to explore this possibility. It is imperative to assess the role of youth police stops in sleep across national contexts such as the UK, considering the distinct features of policing in the UK relative to the USA, as well as the fact that sleep problems among youth remain prevalent in the UK, with one in three youths aged 11–16 years reporting sleep problems (Sadler et al., 2018). In the present study, we employ data from the UK Millennium Cohort Study (MCS) to examine associations between police stops and sleep problems among a national sample of youth.

## 2 METHODS

Data for the present study come from the UK MCS. The MCS is a national, longitudinal study of 18,818 children born in the UK between 2000 and 2002. The MCS data were obtained using a stratified cluster sampling design, with the population being stratified by UK country – England, Wales, Scotland, and Northern Ireland. The study oversampled children from families living in disadvantaged areas (i.e., the poorest 25% of wards from the ward‐based Child Poverty Index) and in those with higher proportions of ethnic minority groups (wards that had an ethnic minority indictor of ≥30%). Thus, the final sample included a higher number of children and families at risk of various forms of adversity and hardship (relative to the general population), which makes the data well‐suited to our research question. Even so, the data contain the appropriate sampling weights to produce findings that are nationally representative and generalisable to the UK as a whole.

To date, data have been collected at seven intervals labelled “Sweeps” (henceforth noted as “S”) when children were aged ~9 months (S1, 2001), 3 years (S2, 2004), 5 years (S3, 2006), 7 years (S4, 2008), 11 years (S5, 2012), 14 years (S6, 2015), and 17 years (S7, 2018). The present study employs data from S5 (aged 11 years) and S6 (aged 14 years) and is restricted to the subsample of youth who participated in the young person questionnaire at S6 and had valid police stop and survey sleep data (*N* = 11,200). For additional details on the study design and sample, see hhtps://cls.ucl.ac.uk.

### 2.1 Sleep problems

#### 2.1.1 Short sleep duration

At S6 (aged 14 years), youth were asked two questions about their wake times and sleep times on school nights: (1) “About what time do you usually go to sleep on a school night?” Responses included *Before 9:00 p.m*. (1), *9:00*–*9*:*59 p.m*. (2), *10:00*–*10*:*59 p.m*. (3), *11:00*–*12:00 p.m*. (4), and *after 12:00 p.m*. (5); (2) “About what time do you usually wake up in the morning on a school day?” Responses included *Before 6:00 a.m*. (1), *6:00*–*6*:*59 a.m*. (2), *7:00*–*7*:*59 a.m*. (3*)*, *8:00*–*8*:*59 a.m*. (4), and *after 9:00 a.m*. (5). We followed the lead of prior research (Hisler et al., 2020) and employed responses to these two items to calculate approximate sleep hours for school nights. Also, in line with Hisler et al. (2020), we considered youth meeting criteria for a short sleep duration if they obtained ≤7 h of sleep. Importantly, this coding strategy was also decided upon in line with the recommendations of the National Sleep Foundation (Hirshkowitz et al., 2015a), which recommends youth in this age group (aged ~14 years) obtain a recommended minimum of 8 h of sleep. In ancillary analyses of a subsample of youth (*N* = 3,827), S6 sleep duration data from daily time‐use diaries (TUDs) was also employed. For more details on this ancillary analysis and its findings, see Appendix A.

#### 2.1.2 Sleep latency

Also at S6, youth were asked the following question about their sleep latency: “During the last 4 weeks, how long did it usually take for you to fall asleep?”. Response options to this item included *0*–*15 min* (1), *16*–*30 min* (2), *31*–*45 min* (3), *46*–*60 min* (4), and *>60 min* (5). Also in line with prior MCS research (Hisler et al., 2020), we dichotomised this item so that youth meeting the criterion for sleep latency problems, designated as >60 min to fall asleep, were assigned a value of 1 and all other youth with valid data were assigned a value of 0.

#### 2.1.3 Mid‐sleep awakenings

Finally, at S6, youth were asked the following question about their mid‐sleep awakenings or sleep disruptions: “During the last 4 weeks, how often did you awaken during your sleep and have trouble falling back to sleep again?”. Responses to the second item ranged from *All of the Time* (1), *Most of the Time* (2), *A Good Bit of the Time* (3), *Some of the Time* (4), *A Little of the Time* (5), and *None of the Time* (6). Also in line with prior MCS research (Hisler et al., 2020), this item was coded dichotomously to identify youth meeting designated criteria for sleep problems on this domain, with youth reporting mid‐sleep awakenings all or most nights being assigned a value of 1 and all other youth being assigned a value of 0.

#### 2.1.4 Composite sleep problems

Finally, we created a composite sleep problems measure, capturing youth reporting *no sleep problems* (0), *a single sleep problem* (1), or *multiple* (i.e., two or more) *sleep problems* (2).

### 2.2 Police contact

To measure police contact in the present study, we followed the lead of prior research (Jackson, Testa, & Boccio, 2022; Jackson, Testa, Fix, et al., 2021). To capture youths’ experiences with police‐initiated contact, youth were asked at S6, “Have you ever been stopped and questioned by the police?” Response options included *Yes* and *No*. In two follow‐up questions, additional details about police stop features pertaining to officer warning/cautions (i.e., “Have you ever been given a formal warning or caution by a police officer?”) and youth arrest (i.e., “Have you ever been arrested by a police officer and taken to a police station?”) were also obtained. Youth who reported any form of lifetime police‐initiated contact were assigned a value of 1; otherwise, they were assigned a value of 0. As in prior MCS research (Jackson, Testa, & Boccio, 2022; Jackson, Testa, Fix, et al., 2021), and in order to examine the robustness of results across features of police contact, details pertaining to officer warnings/cautions and youth arrest were also examined separately from being only stopped and questioned by police in a subset of models.

### 2.3 Covariates

The following covariates were included in multivariate models to minimise the likelihood of spurious results: age, sex (male = 1), race/ethnicity (Asian, Black, Mixed race, and Other race, with White as the reference category), property delinquency (S5; see Staff et al., 2015), substance use (including alcohol and cigarette use, S5; See Staff et al., 2015, 2016), internalising behaviour (the emotional symptoms and peer problems subscales of the Strengths and Difficulties Questionnaire [SDQ], S5; see Bevilacqua et al., 2021), externalising behaviour (the hyperactivity and inattention and conduct problems subscales of the Strengths and Difficulties Questionnaire [SDQ], S5; see Bevilacqua et al., 2021), child attention‐deficit hyperactivity disorder (ADHD) diagnosis (i.e., parent reports of whether a doctor or health professional had ever told the parent that the child had ADHD, S5), parent education (S5) (as measured by National Vocational Qualifications (NVQs), with NVQ 1 as the reference category), household income (S5) (as measured in quintiles, with the first quintile as the reference category), single‐parent household (S5), and low neighbourhood safety (child responses to the question, “How safe is it to walk, play, or hang out in this area during the day?”; higher scores reflect lower neighbourhood safety, S5).

## 3 PLAN OF ANALYSIS

The analysis proceeded as follows. First, we calculated descriptive statistics pertaining to the full analytical sample of youth (*N* = 11,200), stratified by youth police contact. Second, we estimated unadjusted and adjusted logistic regression models of the association between police stops and the three indicators of sleep problems examined in the present study: short sleep duration, sleep latency, and mid‐sleep awakenings. Third, we estimated unadjusted and adjusted multinomial logistic regression models of the association between police stops and the indicator of composite sleep problems. Fourth, we estimated unadjusted and adjusted logistic and multinomial logistic regression models of the association between police stops *features* (i.e., stopped and questioned, warned/cautioned, or arrested) and all sleep measures, including the indicator of composite sleep problems. Finally, a figure was constructed to plot the predicted probabilities of sleep problems by features of police stops. Predicted probabilities were calculated using the marginal standardisation technique, which is a regression‐based form of the common technique of standardisation and is designed to proportionally adjust the estimate of interest according to a weight pertaining to each level of the confounding factors (Muller & MacLehose, 2014). This approach allows predicted probabilities to be employed to make inferences about the whole sample. Ancillary sleep duration analyses of a subsample of youth using TUDs were also performed (*N* = 3,827; for more details, see Appendix A). All analyses were conducted in STATA v 17.1 (StataCorp, College Station, TX, USA) using multiply imputed data (chained equations, 20 imputations) and weights were employed to adjust for nonresponse, probability of selection, and the demographic distribution of the target population.

## 4 RESULTS

First, our findings revealed that 1,779 youth had experienced some form of police‐initiated contact during their lifetime, while 9,421 had not (Table 1). Still, among those with some form of police‐initiated contact, only a small proportion reported arrests (6.02%). In terms of sleep problems, 12.82% reported short sleep durations (≤7 h), 9.83% reported sleep latency of >60 min, and 10.94% reported mid‐sleep awakenings on most or all nights of the week. Notably, the prevalence of these sleep problems differed significantly by police contact. For instance, while only 11.28% of youth reporting no police‐initiated contact reported short sleep durations, nearly twice as many (20.97%) youths experiencing police‐initiated contact reported short sleep durations. Many covariates also differ significantly by police contact. To highlight a few, youth reporting police contact were significantly more likely to be male and older, but significantly less likely to be Asian (compared to White). Additionally, youth with police contact were significantly more likely to report a history of substance use and property offending, as well as higher levels of externalising behaviour and an ADHD diagnosis. Overall, youth experiencing police contact also reported parents with lower levels of education and households with lower incomes. Finally, youth reporting police contact were also significantly more likely to reside in a single‐parent household and report lower levels of neighbourhood safety (for more details, see Table 1).

**TABLE 1:** Descriptive statistics stratified by police stops at age 14 years (S6) (*N* = 11,200)

Police stops			
	No (*N* = 9,421)	Yes (*N* = 1,779)	*t* statistic
Sleep problems, %			
Short sleep duration, %	11.28	20.97	11.27**
Sleep latency, %	9.06	13.88	6.27**
Mid‐sleep awakenings, %	9.70	17.54	9.75**
Composite	0.30	0.52	13.68**
Police stop features, %			
Stopped and questioned	–	52.16	–
Warned and cautioned	–	41.82	–
Arrested/taken into custody	–	6.02	–
Covariates			
Age, years (S6)	13.76	13.81	4.36**
Male, %	46.98	61.50	11.29**
Race/ethnicity: White^a^, %	79.42	83.02	3.49**
Race/ethnicity: Asian, %	11.80	5.62	−7.72**
Race/ethnicity: Black, %	3.11	3.37	0.58
Race/ethnicity: Mixed, %	4.45	6.13	3.07**
Race/ethnicity: Other, %	1.22	1.85	2.15*
Property delinquency (S5), %	5.57	18.72	19.43**
Substance use (S5), %	10.30	25.18	17.55**
Internalising behaviour (S5), SDQ score	1.31	1.33	3.31**
Externalising behaviour (S5), SDQ score	1.41	1.58	19.97**
ADHD diagnosis (S5), %	0.79	2.08	5.06**
Parent education: NVQ 1 (S5)^a^, %	5.70	8.60	4.67**
Parent education: NVQ 2 (S5), %	22.41	28.89	5.93**
Parent education: NVQ 3 (S5), %	14.71	15.96	1.36
Parent education: NVQ 4 (S5), %	35.76	25.52	−8.38**
Parent education: NVQ 5 (S5), %	9.92	6.80	−4.14**
Parent education: overseas/other education (S5), %	3.00	3.26	0.60
Parent education: none (S5), %	8.54	10.96	3.28**
Household income: first quintile (S5)^a^, %	15.57	25.41	10.14**
Household income: second quintile (S5), %	17.20	23.61	6.44**
Household income: third quintile (S5), %	20.89	22.54	1.56
Household income: fourth quintile (S5), %	23.32	16.81	−6.06**
Household income: fifth quintile (S5), %	23.04	11.64	−10.85**
Single‐parent household (S5), %	19.80	30.10	11.60**
Low neighbourhood safety (S5), score	1.81	1.86	2.88*

Abbreviations: ADHD, attention‐deficit hyperactivity disorder; NVQ, National Vocational Qualifications; SDQ, Strengths and Difficulties Questionnaire.

^a^Reference category.

**p < 0.01.

*p < 0.05.

Our multivariate findings, shown in Table 2, also reveal a largely consistent pattern in which police stops significantly elevated the odds of short sleep durations (odds ratio [OR] 2.09, 95% confidence interval [CI] 1.83–2.37), sleep latency of >60 min (OR 1.62, 95% CI 1.39–1.88), and frequent mid‐sleep awakenings (OR 1.98, 95% CI 1.72–2.28). Importantly, these associations were robust to the inclusion of covariates, despite slight attention in point estimates. Multivariate models revealed that, beyond police contact, the most consistently significant predictors of each of the three sleep problem indicators were sex (with male youth reporting significantly lower odds of each sleep problem) and the third quintile or above household income (with youth from wealthier households reporting lower odds of each sleep problem). Notably, child substance use significantly predicted both a shorter sleep duration and longer sleep latency, whereas both internalising and externalising behaviour significantly predicted longer sleep latency and more frequent mid‐sleep awakenings. Furthermore, among the racial/ethnic groups, Asian youth were significantly less likely to report short sleep durations or long sleep latency relative to White youth.

**TABLE 2:** The association between police stops and sleep problems among youth in the UK (*N* = 11,200)

	Short Sleep Duration		Sleep latency		Mid‐sleep awakenings	
	OR (95% CI)	AOR (95% CI)	OR (95% CI)	AOR (95% CI)	OR (95% CI)	AOR (95% CI)
Stopped by police	2.09** (1.83–2.37)	1.78** (1.54–2.05)	1.62** (1.39–1.88)	1.42** (1.21–1.67)	1.98** (1.72–2.28)	1.69** (1.44–1.96)
Covariates						
Age, years (S6)	*–*	1.23** (1.08–1.40)	–	1.04 (0.90–1.19)	–	1.06 (0.93–1.22)
Male	*–*	0.69** (0.62–0.78)	–	0.76** (0.66–0.86)	–	0.49** (0.43–0.56)
Race/ethnicity: Asian	*–*	0.77* (0.61–0.97)	–	0.64** (0.50–0.83)	–	1.03 (0.83–1.27)
Race/ethnicity: Black	*–*	1.07 (0.78–1.47)	–	0.93 (0.65–1.35)	–	1.19 (0.86–1.65)
Race/ethnicity: Mixed	*–*	1.12 (0.87–1.44)	–	1.08 (0.81–1.43)	–	1.07 (0.81–1.42)
Race/ethnicity: Other	*–*	0.85 (0.51–1.39)	–	0.64 (0.34–1.19)	–	1.05 (0.65–1.70)
Property delinquency (S5)	*–*	1.31* (1.05–1.63)	–	1.17 (0.93–1.48)	–	1.20 (0.95–1.50)
Substance use (S5)	*–*	1.42** (1.21–1.67)	–	1.30** (1.08–1.57)	–	1.13 (0.92–1.38)
Internalising behaviour (S5)	*–*	1.16 (0.94–1.46)	–	1.48** (1.18–1.86)	–	1.36* (1.06–1.75)
Externalising behaviour (S5)	*–*	1.19 (0.98–1.46)	–	1.26* (1.02–1.56)	–	1.82** (1.48–2.24)
ADHD diagnosis (S5)	*–*	0.90 (0.48–1.67)	–	1.70 (0.91–3.16)	–	1.48 (0.91–2.41)
Parent education: NVQ 2 (S5)	*–*	1.04 (0.81–1.33)	–	1.16 (0.87–1.56)	–	1.19 (0.90–1.57)
Parent education: NVQ 3 (S5)	*–*	1.08 (0.82–1.42)	–	1.35 (0.99–1.83)	–	1.30 (0.97–1.73)
Parent education: NVQ 4 (S5)	*–*	1.02 (0.79–1.33)	–	1.28 (0.95–1.73)	–	1.20 (0.91–1.59)
Parent education: NVQ 5 (S5)	*–*	1.06 (0.76–1.47)	–	1.42 (0.98–2.06)	–	1.41 (0.99–2.00)
Parent education: overseas/other (S5)	*–*	1.02 (0.69–1.50)	–	0.87 (0.54–1.41)	–	0.90 (0.56–1.45)
Parent education: none (S5)	*–*	0.90 (0.67–1.22)	–	0.95 (0.66–1.37)	–	1.29 (0.94–1.77)
Household income: second quintile (S5)	*–*	0.88 (0.73–1.05)	–	0.76* (0.60–0.95)	–	0.86 (0.71–1.05)
Household income: third quintile (S5)	*–*	0.81* (0.66–0.99)	–	0.75* (0.59–0.95)	–	0.70** (0.56–0.87)
Household income: fourth quintile (S5)	*–*	0.74* (0.58–0.93)	–	0.68** (0.52–0.89)	–	0.63** (0.49–0.80)
Household income: fifth quintile (S5)	*–*	0.57** (0.44–0.75)	–	0.59** (0.45–0.79)	–	0.41** (0.31–0.55)
Single‐parent household (S5)	*–*	1.03 (0.88–1.20)	–	0.87 (0.73–1.04)	–	1.08 (0.92–1.27)
Low neighbourhood safety (S5)	*–*	0.96 (0.85–1.02)	–	1.05 (0.95–1.17)	–	1.11* (1.01–1.22)

Abbreviations: CI, confidence Interval; NVQ, National Vocational Qualifications; (A)OR, (adjusted) odds ratio.

Reference category for composite sleep problems is “none”. For race/ethnicity, White is the reference category; for parent education (S5), NVQ 1 is the reference category; for household income (S5), the first quintile as the reference category.

*p < 0.05.

**p < 0.01.

In Table 3, multivariate findings also reveal that police stops were more strongly associated with multiple sleep problems (relative risk ratio [RRR] 2.63, 95% CI 2.21–3.13) than a single sleep problem (RRR 1.81, 95% CI 1.61–2.04). These findings, while slightly attenuated, hold after adjustment for confounders (single sleep problem: RRR 1.51, 95% CI 1.35–1.75; multiple sleep problems: RRR 2.17, 95% CI 1.79–2.62). Notably, of the covariates examined, some of the most robust predictors of multiple sleep problems included early substance use (RRR 1.53, 95% CI 1.22–1.92), internalising behaviour (RRR 1.54, 95% CI 1.15–2.06), and externalising behaviour (RRR 1.50, 95% CI 1.15–1.95), in addition to sex (male youth incurred lower risk of multiple sleep problems) and high household income (youth from households with higher incomes incurred lower risk of multiple sleep problems). Notably, as shown in Table 4, the association between police stops and sleep problem indicators largely holds across features of police stops. Analysis of police stop features revealed that, while being stopped and questioned by police was significantly predictive of most, but not all, sleep problems after adjusting for covariates, being warned/cautioned by the police or being arrested was consistently and robustly associated with all sleep problems. Still, the final composite model reveals that the risk of multiple sleep problems increased by 115% among stopped and questioned youth (RRR 2.15, 95% CI 1.70–2.71), 191% among warned/cautioned youth (OR 2.91, 95% CI 2.28–4.36), and 544% among youth arrested/taken into custody (OR 6.44, 95% CI 3.81–10.90). Analyses using continuous sleep outcomes produced similar findings (see Appendix B), and short sleep duration results from analyses using TUDs among a subsample of youth corroborate these patterns (see Appendix A).

**TABLE 3:** The association between police stops and composite sleep problems among youth in the UK (*N* = 11,200)

	Composite sleep problems			
	Single		Multiple	
	RRR (95% CI)	ARRR (95% CI)	RRR (95% CI)	ARRR (95% CI)
Stopped by police	1.81** (1.61–2.04)	1.51** (1.35–1.75)	2.63** (2.21–3.13)	2.17** (1.79–2.62)
Covariates				
Age, years (S6)	–	1.17** (1.05–1.30)	–	1.09 (0.91–1.29)
Male	–	0.72** (0.65–0.80)	–	0.46** (0.39–0.55)
Race/ethnicity: Asian	–	0.96 (0.80–1.15)	–	0.59** (0.43–0.82)
Race/ethnicity: Black	–	1.13 (0.86–1.47)	–	0.98 (0.63–1.53)
Race/ethnicity: Mixed	–	1.41** (1.41–1.74)	–	0.93 (0.64–1.36)
Race/ethnicity: Other	–	1.01 (0.68–1.52)	–	0.62 (0.30–1.31)
Property delinquency (S5)	–	1.29* (1.06–1.57)	–	1.28 (0.96–1.71)
Substance use (S5)	–	1.26** (1.08–1.47)	–	1.53** (1.22–1.92)
Internalising behaviour (S5)	–	1.24* (1.03–1.50)	–	1.54** (1.15–2.06)
Externalising behaviour (S5)	–	1.58 (1.34–1.87)	–	1.50** (1.15–1.95)
ADHD diagnosis (S5)	–	0.99 (0.60–1.63)	–	1.87 (0.98–3.59)
Parent education: NVQ 2 (S5)	–	1.17 (0.94–1.47)	–	1.19 (0.86–1.65)
Parent education: NVQ 3 (S5)	–	1.22 (0.95–1.55)	–	1.39 (0.96–1.99)
Parent education: NVQ 4 (S5)	–	1.21 (0.95–1.53)	–	1.20 (0.85–1.70)
Parent education: NVQ 5 (S5)	–	1.39* (1.04–1.86)	–	1.32 (0.84–2.09)
Parent education: overseas/other (S5)	–	1.04 (0.73–1.48)	–	0.89 (0.50–1.58)
Parent education: None (S5)	–	1.19 (0.92–1.54)	–	1.00 (0.67–1.48)
Household income: second quintile (S5)	–	0.94 (0.80–1.10)	–	0.71** (0.54–0.91)
Household income: third quintile (S5)	–	0.84 (0.71–1.00)	–	0.59** (0.45–0.78)
Household income: fourth quintile (S5)	–	0.73** (0.60–0.89)	–	0.54** (0.40–0.74)
Household income: fifth quintile (S5)	–	0.60** (0.48–0.74)	–	0.36** (0.25–0.52)
Single‐parent household (S5)	–	1.03 (0.90–1.18)	–	0.99 (0.80–1.20)
Low neighbourhood safety (S5)	–	1.02 (0.95–1.10)	–	1.04 (0.91–1.18)

Abbreviations: ADHD, attention‐deficit hyperactivity disorder; CI, confidence interval; NVQ, National Vocational Qualifications; (A)RRR, (adjusted) relative risk ratio.

For race/ethnicity, White is the reference category; for Parent Education (S5), NVQ 1 is the reference category; for Household Income (S5), the first quintile as the reference category.

*p < 0.05.

**p < 0.01.

**TABLE 4:** The association between police stop features and sleep problems among youth in the UK (*N* = 11,200)

Composite sleep problems										
	Short sleep duration		Sleep latency		Mid‐sleep awakenings		Single		Multiple	
	OR (95% CI)	AOR (95% CI)	OR (95% CI)	AO (95% CI)	OR (95%CI)	AOR (95%CI)	RRR (95% CI)	ARRR (95% CI)	RRR (95% CI)	ARRR (95% CI)
Police stop features										
Stopped and questioned	1.87** (1.57–2.22)	1.67** (1.39–2.01)	1.29* (1.04–1.60)	1.21 (0.97–1.50)	1.82** (1.51–2.20)	1.71** (1.40–2.08)	1.53** (1.30–1.80)	1.39** (1.17–1.64)	2.15** (1.70–2.71)	1.94** (1.52–2.47)
Warned/cautioned	2.19** (1.82–2.64)	1.82** (1.49–2.22)	1.85** (1.50–2.29)	1.59** (1.27–1.98)	1.97** (1.61–2.41)	1.57** (1.26–1.95)	1.99** (2.67–2.36)	1.62** (1.35–1.95)	2.91** (2.28–4.36)	2.27** (1.75–2.95)
Arrested/taken into custody	3.50** (2.31–5.30)	2.54** (1.64–3.91)	3.05** (1.94–4.80)	2.29** (1.43–3.68)	3.62** (2.36–5.55)	2.31** (1.46–3.65)	3.86** (2.52–5.93)	2.67** (1.71–4.15)	6.44** (3.81–10.90)	4.11** (2.36–7.15)

CI, confidence interval; (A)OR, (adjusted) odds ratio; (A)RRR, (adjusted) relative risk ratio.

The following covariates are included in each adjusted model but suppressed to conserve space: Age, Sex, Race/Ethnicity (Asian, Black, Mixed race, and Other race, with White as the reference category), Property Delinquency (S5), Substance Use (S5), Internalising Behaviour (S5), Externalising Behaviour (S5), attention‐deficit hyperactivity disorder (ADHD) Diagnosis (S5), Parent Education (S5) (as measured by National Vocational Qualifications [NVQs], with NVQ 1 as the reference category), Household Income (S5) (as measured in quintiles, with the first quintile as the reference category), Single‐Parent Household (S5), and Low Neighbourhood Safety (S5).

*p < 0.05.

**p < 0.01.

**FIGURE 1:**
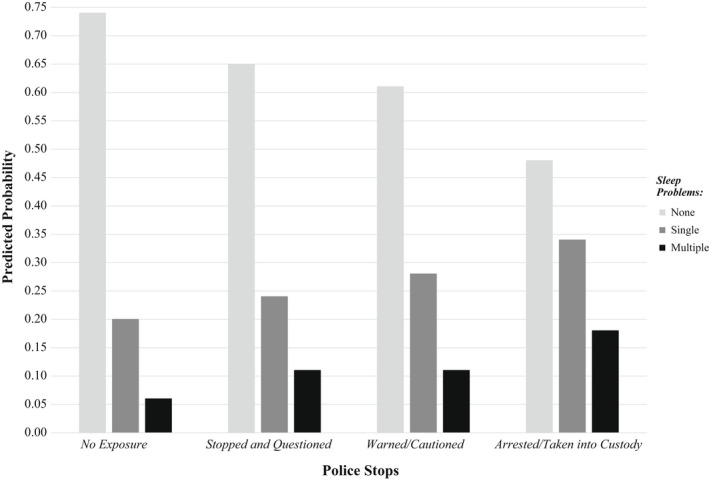
Predicted probability of youth sleep problems by police stop exposure and features using the marginal standardisation technique

Finally, Figure 1 illustrates that the predicted probability of *no sleep problems* among youth reporting no lifetime police‐initiated contact was 0.74. This suggests that, even after adjustment for covariates, about three in four youth with no police‐initiated contact experience no sleep problems. By comparison, the predicted probability of *no sleep problems* among youth who have been arrested/taken into custody was 35% lower (0.48), with less than half of youth experiencing arrest predicted to have no sleep problems. In contrast, the predicted probability of multiple sleep problems was nearly one in five among arrested youth (0.18), but fewer than one in 16 (0.06) among youth with no police‐initiated contact. Youth with other police exposures, such as stopped and questioned or warned/cautioned, also exhibited a greater predicted probability of multiple sleep problems than those with no stop, despite having a lower predicted probability than arrested youth.

## 5 DISCUSSION

The present study provides the first assessment of the association between police contact and sleep among youth in the UK. The findings reveal youth–police contact was significantly associated with shorter sleep durations, longer sleep latency, and more frequent mid‐sleep awakenings. Ancillary analyses using TUDs largely corroborate these findings in the case of short sleep durations. Furthermore, the odds of experiencing multiple sleep problems were greatest among youth who were arrested/taken into custody, but significant nonetheless even in the absence of arrest. These results echo the limited United States‐based research on how police contact might worsen sleep among adolescents (Jackson et al., 2020) and adults (Testa et al., 2021).

We note a few limitations to be expanded upon in future research. First, the measures of sleep were self‐reported (including ancillary TUD‐based analyses) and therefore may be subject to more measurement error relative to objectively measured sleep (i.e., polysomnography, actigraphy; see Van de Water et al., 2011). Still, across two different data collection strategies (i.e., surveys versus TUDs), the results were largely consistent. Second, the MCS data lack some details about the context of the police stop that could be valuable to understanding the connection between police contact and sleep in the UK, including officer intrusiveness, stop location, and youth perceptions of procedural justice (Jackson et al., 2020). The extant, United States‐based research on this topic suggests that these details can be important when examining indicators of sleep quality (e.g., sleep latency, mid‐sleep awakenings). Finally, because of the possibility of omitted variable bias, the findings should be considered associational rather than causal. Relatedly, the present study cannot speak to the precise process by which police stops might influence downstream sleep patterns, despite our inclusion of models adjusting for pre‐existing risk factors for police contact and sleep problems (e.g., behavioural and mental health factors). Future research should certainly seek to tease apart this process further. However, extant research suggests that the connection between police contact and youth sleep problems may be, at least partially, a function of what more intrusive, potentially distressing contacts can yield in terms of feelings of social stigma and even post‐traumatic stress (Jackson et al., 2020). While we are unable to replicate those precise details in the present study, our finding that stops featuring either an arrest or a warning/caution are particularly consequential for youth sleep in the UK is consistent with the United States‐based findings pointing to stops characterised by officer treatment perceived as unfair or intrusive by citizens as most consequential (Jackson et al., 2020; Testa et al., 2021).

With these limitations in mind, the findings suggest potential avenues to improve the sleep of youth with police contact. Considering the growing evidence that police contact is adversely associated with both psychological wellbeing (Geller et al., 2014; Jackson et al., 2019; McFarland et al., 2019; Turney, 2021) and sleep (Jackson et al., 2020; Testa et al., 2021), these findings suggest that stopped youth might benefit from referrals to medical practitioners or trained mental health counsellors who can provide support to ameliorate any psychological ramifications that might impair sleep, such as post‐traumatic stress, hyperarousal, and rumination (Bader et al.,. 2007). Relatedly, it may be useful for medical providers and school counsellors to screen for past experiences with police contact to connect youth with appropriate school‐based and community supports and resources. These providers might also consider the possibility that youth screening positive for sleep problems in heavily policed communities might need additional trauma‐informed supports to adequately treat their sleep needs.

Additionally, integrating more trauma‐informed policing (TIP) approaches into the UK policing model may reduce the negative consequences of youth–police contact. Specifically, TIP encourages police to recognise signs of trauma stemming from a police officer–citizen encounter and provide police agencies with training and resources to mitigate such trauma. Under this model, police can work in teams with schools and health professionals to better recognise signs of trauma, as well as implement strategies to support youth wellbeing after a trauma‐inducing event (Gill et al., 2016).

## 6 CONCLUSIONS

Using a representative sample of adolescents in the UK, this study finds evidence that youth–police contact is associated with greater sleep problems across indicators. This study provides the first evidence of a connection between police contact and sleep problems outside of the USA and reiterates the need to focus on the diverse health repercussions of youth–police contact across national contexts.

## CONFLICTS OF INTEREST

None.

## AUTHOR CONTRIBUTIONS

Dylan B. Jackson conceptualised and designed the study, conducted the statistical analyses, interpreted the results, and drafted sections of the manuscript. Alexander Testa advised on statistical analyses and design of the study, and drafted sections of the manuscript. All authors approved the final manuscript as submitted.

## FUNDING INFORMATION

No funding to declare.

## Data Availability

Data openly available in a public repository.

## APPENDIX A

### Daily Time‐Use Diary Sleep Data

At Sweep 6, when youth were 14 years of age, a subsample of youth completed time‐use diaries (TUDs), which included information about the time they spent doing a variety of activities in real time (including sleep and 43 other pre‐coded activities). TUDs were completing at 10‐min intervals throughout the day, with the present analyses being restricted to youth who had valid sleep data derived from a weekday TUD (Monday–Friday) and a weekend TUD (Saturday or Sunday). Participants completed TUDs using one of three methods: online (entered in web form), app (entered in a smartphone or tablet application), and paper (entered in paper form and submitted to research team manually), with online/digital forms being strongly encouraged. Participants received text message reminders to complete TUDs (to which they consented) on the day before and the morning of each TUD. The 24‐h periods ran from 4:00 a.m. on the selected day to 4:00 a.m. the next day. As expected, sleep occupied about a third of entries, on average, despite substantial variation.

For the present study, we identified sleep durations by flagging the sleep code across each 10‐min TUD slot for each youth – on their selected weekday TUD, as well as their selected weekend TUD. Youth who did not participate in both weekend and weekday TUDs were excluded from the analysis, resulting in a sample of 3,844 youth. An additional 17 youth with an ADHD diagnosis at S5 was also dropped. The subsample of children diagnosed with ADHD at S5 (*N* = 17) were removed from the sample for this analysis, as inclusion of ADHD diagnosis as a covariate led to highly unstable estimates given the rarity of a diagnosis (0.44% of this subsample). As was the case with the main analyses, missing data on covariates were multiply imputed using STATA 17.1. As was the case in the main analysis, and in line with guidelines from the National Sleep Foundation (Hirschkowitz et al., 2015), youth whose sleep duration was shorter than the recommended duration for their age group (i.e., ≤7 h for 14‐year‐old youth) were designated as experiencing short sleep durations (20.81%). Notably, analyses employing shorter sleep durations (e.g., <7 h of sleep; <6 h of sleep) did not meaningfully alter the results.

The association between police stops, police stop features, and short sleep duration among youth in the UK using time‐use diary (TUD) Data (*N* = 3,827)

**Table:**

Short sleep duration (≤7 h)		
	OR (95% CI)	AOR (95% CI)
Stopped by police	1.47** (1.17–1.85)	1.39** (1.10–1.75)
Police stop features		
Stopped and questioned	1.35* (1.01–1.80)	1.29 (0.96–1.73)
Warned/cautioned	1.57** (1.10–2.24)	1.46* (1.02–2.11)
Arrested/taken into custody	3.55* (1.14–11.02)	3.05* (1.02–9.67)

CI, confidence interval; (A)OR, (adjusted) odds ratio.

The following covariates are included in each adjusted model but suppressed to conserve space: Age, Sex, Race/Ethnicity (Asian, Black, Mixed race, and Other race, with White as the reference category), Property Delinquency (S5), Substance Use (S5), Internalising Behaviour (S5), Externalising Behaviour (S5), Parent Education (S5) (as measured by National Vocational Qualifications [NVQs], with NVQ 1 as the reference category), Household Income (S5) (as measured in quintiles, with the first quintile as the reference category), Single‐Parent Household (S5), and Low Neighbourhood Safety (S5). Models also adjusted for method of TUD completion (online, app, paper). The subsample of children diagnosed with ADHD at S5 (*N* = 17) were removed from the sample for this analysis, as inclusion of ADHD diagnosis as a covariate led to highly unstable estimates given the rarity of a diagnosis (0.44% of this subsample).

**p* < 0.05.

***p* < 0.01.

## APPENDIX B

The association between police stops, police stop features, and sleep among youth (*N* = 11,200) using continuous outcomes

**Table:**

	Short sleep duration	Sleep latency	Mid‐sleep awakenings
	B (SE)	B (SE)	B (SE)
Stopped by police	0.30** (0.03)	0.21** (0.03)	0.38** (0.04)
Police stop features			
Stopped and questioned	0.27** (0.03)	0.15** (0.04)	0.39** (0.05)
Warned/cautioned	0.32** (0.04)	0.24** (0.05)	0.33** (0.05)
Arrested/taken into custody	0.36** (0.09)	0.57** (0.12)	0.64** (0.13)

B, unstandardised coefficient; SE, standard error.

The following covariates are included in all models but are suppressed to conserve space: Age, Sex, Race/Ethnicity (Asian, Black, Mixed race, and Other race, with White as the reference category), Property Delinquency (S5), Substance Use (S5), Internalising Behaviour (S5), Externalising Behaviour (S5), ADHD Diagnoses (S5), Parent Education (S5) (as measured by National Vocational Qualifications [NVQs], with NVQ 1 as the reference category), Household Income (S5) (as measured in quintiles, with the first quintile as the reference category), Single‐Parent Household (S5), and Low Neighbourhood Safety (S5).

**p* < 0.05.

***p* < 0.01.
